# Pedicle frozen autograft–prosthesis composite reconstructions for malignant bone tumors of the proximal femur

**DOI:** 10.1186/s12891-020-3112-0

**Published:** 2020-02-06

**Authors:** Gang Xu, Shinji Miwa, Norio Yamamoto, Katsuhiro Hayashi, Akihiko Takeuchi, Kentaro Igarashi, Takashi Higuchi, Yuta Taniguchi, Yoshihiro Araki, Hirotaka Yonezawa, Sei Morinaga, Hiroyuki Tsuchiya

**Affiliations:** Department of Orthopaedic Surgery, Kanazawa University School of Medicine, 13-1 Takara-machi, Kanazawa, Ishikawa 920-8641 Japan

**Keywords:** Frozen autograft–prosthesis composite, Proximal femur, Malignant bone tumors, Biological reconstruction, Tumor–bearing bone graft, Liquid nitrogen

## Abstract

**Background:**

Limb salvage surgery is becoming increasingly popular after tumor resection in the lower extremity. Biological reconstruction and use of megaprosthesis are main methods for malignant bone tumors of the proximal femur, which remain controversial due to short- and long-term complication in the proximal femur. Tumor-bearing bone treated by liquid nitrogen is one of biological reconstruction. This study aimed to evaluate the mid- and long-term functional outcomes and complications in patients treated with frozen autograft–prosthesis composite (FAPC) reconstructions in the proximal femur.

**Methods:**

This retrospective study included 19 patients (10 women, 9 men) with malignant tumors of the proximal femur who underwent tumor-wide resection and FAPC reconstruction (mean age, 46 years; range, 9–77 years). The mean follow-up period of 69 months (range, 9–179 months). Functional outcomes, oncological outcome and complications were evaluated by Musculoskeletal Tumor Society score, clinical and radiological examinations.

**Results:**

The overall survival rate was 68.4%, and the mean Musculoskeletal Tumor Society functional score was 26.4 points (88%). FAPC survival rates were 100 and 50% at 5 and 10 years, respectively. Five of the 19 patients (26%) had complications: 2 required prosthesis removal and 2 developed a deep infection around acetabular. Wear of the acetabulum occurred in 2 cases, while disease recurrence was occurred in 1 case. There were no cases of greater trochanter avulsion, obvious absorption around frozen bone, prosthesis loosening or leg length discrepancy.

**Conclusions:**

Due to without femoral osteotomy, this technique features satisfactory functional outcome and provide biomechanical stability that is comparable to those of other methods of biological reconstruction or megaprosthesis.

## Introduction

The proximal femur is a common site of malignant bone tumors, however, the options for reconstruction after tumor resection are limited due to the requirement of reconstruction of the hip-joint. In recent years, the quality of the patient’s life has been remarkably improved largely attributed to the great advances in chemotherapy, radiotherapy, radiological examination and surgical techniques. The 5-year survival rate of non-metastatic osteosarcoma has increased to close to 70%, while the lower-extremity functional outcomes were 71–87% on the Musculoskeletal Tumor Society (MSTS) system [1, 2]. Importantly, no significant difference was reported in overall survival rate or functional outcome between amputation and limb-sparing surgery [3]. Therefore, limb salvage surgery is becoming increasingly popular after tumor resection in the lower extremity [2–4].

Megaprosthesis and allograft–prosthesis composite (APC) reconstructions are the most widely used in the proximal femur reconstruction [4, 5]. Megaprosthesis and APC reportedly have various advantages in short- and long-term functional outcomes, respectively [4, 6, 7]. However, the biological and nonbiological reconstructions after malignant tumor resection remain controversial in the proximal femur. In principle, the ideal proximal femur reconstruction method includes local tumor control, optimal limb function stability, and restoration of the maximal hip abductor function. Since 1999, we developed a tumor-bearing autograft using liquid nitrogen and reported its application for reconstruction in patients with malignant bone tumor [8]. Pedicle freezing is a method of creating a tumor-bearing autograft using liquid nitrogen [9].

In the current study, we focused on the radiological and functional outcomes for frozen autograft–prosthesis composite (FAPC) reconstruction by pedicle freezing in the proximal femur. We attempted to answer following questions: (1) investigate the mid- and long-term functional outcomes and complications in patients treated with FAPC reconstruction and (2) compare the functional outcomes and survival rates of various reconstruction methods.

## Materials and methods

### Patients

This retrospective study included a total of 23 consecutive patients with malignant tumors of the proximal femur who underwent tumor-wide resection and FAPC reconstruction between 2003 and 2015 at Kanazawa University Hospital. The follow-up period for each patient was > 6 months. Four patients were excluded from this study: 1 patient with incomplete data; 1 patient underwent a freezing operation of the total femur; and 2 patients with follow-up period was < 6 months. Eventually, 19 patients (10 women, 9 men) were included in this study **(**Table 1). The mean patient age was 46 years (range, 9–77 years), while the mean follow-up period was 69 months (range, 9–179 months). Pathological diagnoses included osteosarcoma in 8 patients, chondrosarcoma in 3 patients, undifferentiated pleomorphic sarcoma in 1 patient, and metastatic tumor in 7 patients (breast cancer in 3, lung cancer in 1, renal cell carcinoma in 2, and hepatocellular carcinoma in 1). New-adjuvant and adjuvant chemotherapy were administrated to12 patients. This retrospective study was approved by the ethics committee of Kanazawa University.

**Table 1:** Date and outcomes of patients treated by frozen autograft–pedicle composite in the proximal femur

Case	Diagnosis	Enneking stage	Head articulation	Cement	CTX	Insert LN length (mm)	Stem length in femur (mm)	MSTS Score	Follow-up (mon)	Oncological outcome	Complication/Treatment (mon)
1	Chondrosarcoma	Ib	Bipolar	Yes	No	160	230	20	99	NED	
2	Chondrosarcoma	Ib	Bipolar	Yes	No	270	335	30	144	CDF	Infection with acetabulum/ acetabulum revision (111 m)
3	Chondrosarcoma	Ib	Bipolar	Yes	No	130	290	27	149	CDF	
4	Osteosarcoma	III	Bipolar	Yes	Yes	90	180	30	84	NED	
5	Osteosarcoma	IIb	Bipolar	Yes	Yes	190	290	30	120	CDF	Dislocation/closed reductions
6	Osteosarcoma	IIb	Bipolar	Yes	Yes	150	280	28	24	DOOD	Died of colon cancer(24 m)
7	Osteosarcoma	III	Bipolar	Yes	Yes	50	100	20	44	DOD	
8	Osteosarcoma	III	Bipolar	Yes	Yes	90	145	30	22	DOD	
9	Osteosarcoma	IIa	Bipolar	Yes	Yes	100	140	26	179	NED	Soft tissue recurrence(107 m), wear of the acetabulum(97 m)/ hip disarticulation (107 m)
10	Osteosarcoma	III	Bipolar	Yes	Yes	250	290	23	9	DOD	
11	Osteosarcoma	IIb	Bipolar	Yes	Yes	100	145	24	9	DOD	
12	UPS	IIa	Bipolar	Yes	No	150	210	19	66	CDF	
13	Breast cancer	N/A	Bipolar	Yes	No	110	220	28	57	AWD	
14	Breast cancer	N/A	Bipolar	Yes	No	100	205	27	150	CDF	Fracture (12 m), prothesis fracture(112 m), wear of the acetabulum(106 m)/ megaprosthesis conversion(112 m)
15	Breast cancer	N/A	Bipolar	Yes	Yes	130	180	25	9	AWD	
16	Lung CA	N/A	Bipolar	Yes	Yes	150	280	30	36	NED	
17	RCC	N/A	Bipolar	Yes	No	100	240	30	61	CDF	
18	RCC	N/A	Bipolar	Yes	Yes	110	240	28	33	DOD	
19	HCC	N/A	Bipolar	Yes	Yes	200	250	26	12	AWD	Infection with acetabulum (3 m)
						138	224	26.4	68.8		

*F* female, *M* male, *UPS* undifferentiated pleomorphic sarcoma, *CA* cancer, *RCC* renal cell carcinoma, *HCC* hepatocellular carcinoma, *AWD* alive with disease, *CDF* continuous disease free, *DOD* dead of disease, *DOOD* dead of other disease, *NED* no evidence of disease, *CTX* chemotherapy, *OP* operation, *MSTS* Musculoskeletal Tumor Society

### Surgical procedure and postoperative management

**Fig. 1:**
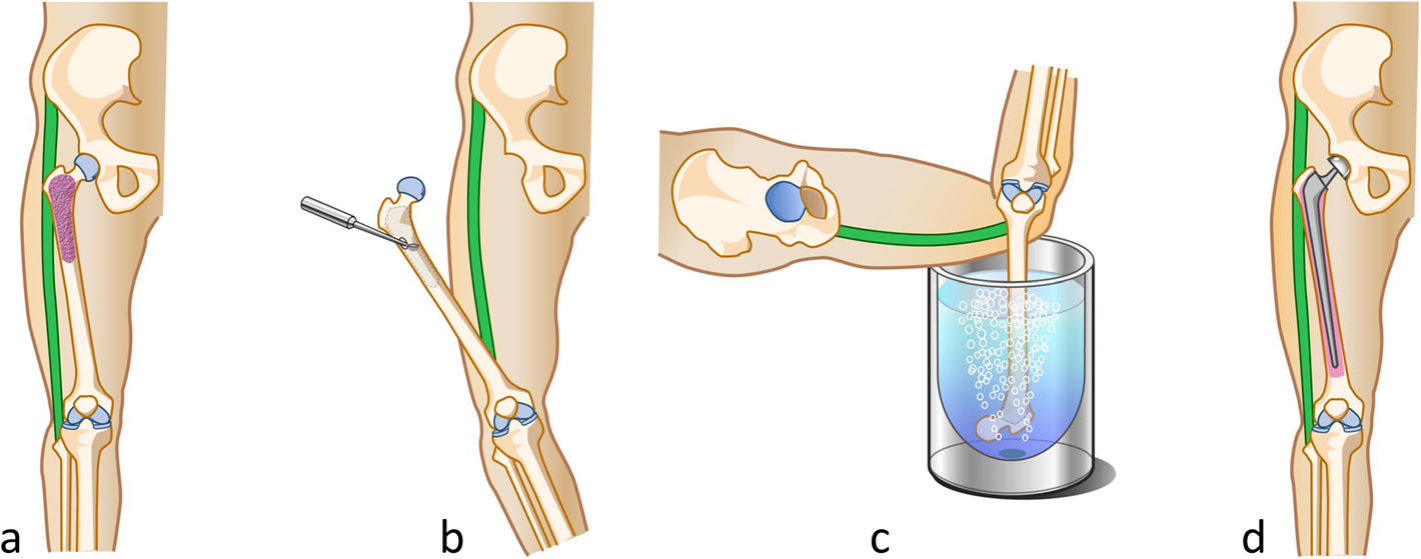
Frozen autograft–prothesis composite used for femoral reconstruction. **a** Tumor in the proximal femur. **b** Curettage of the tumor after joint dislocation. **c** Pedicle freezing in liquid nitrogen. **d** ligament and FAPC reconstruction

**Fig. 2:**
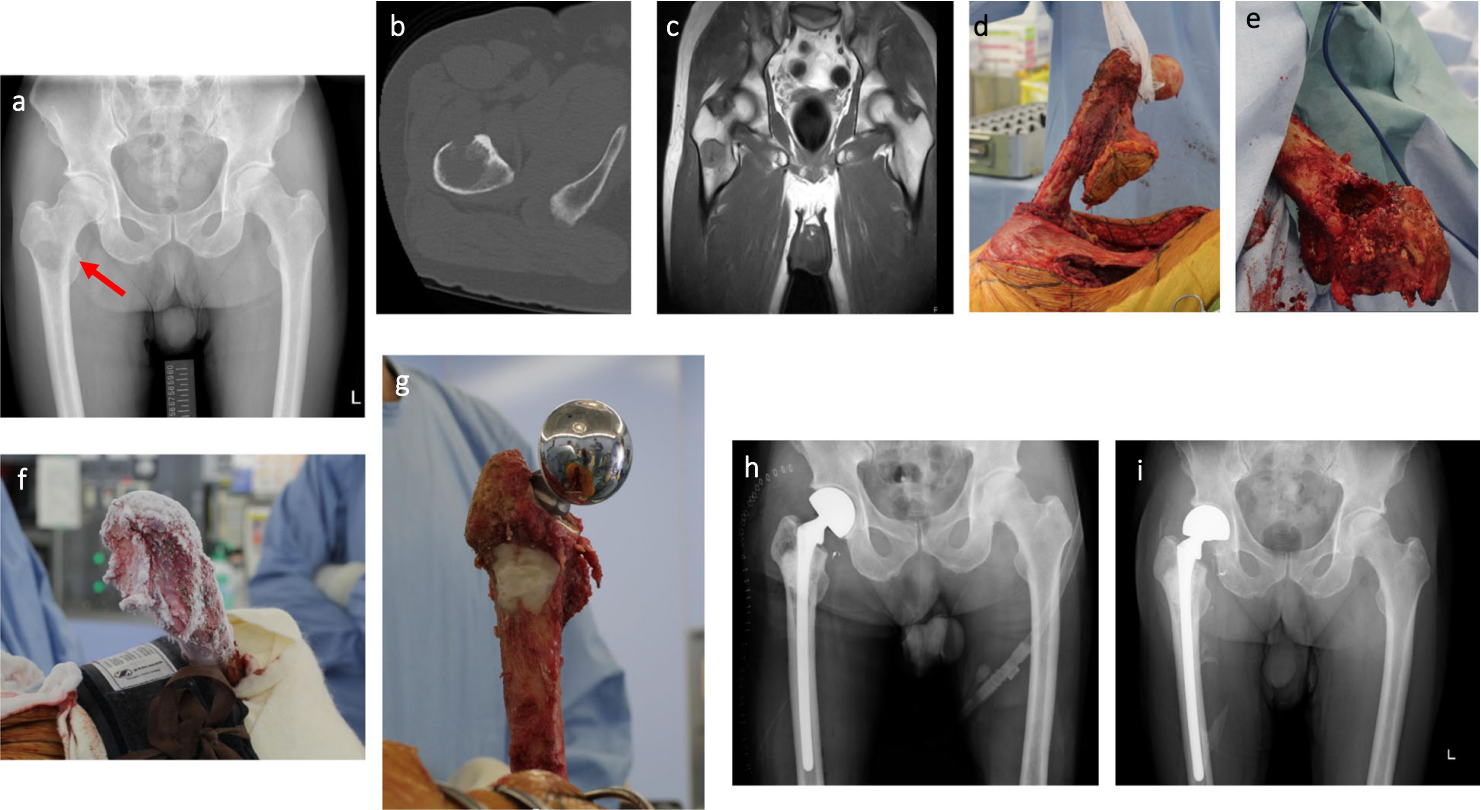
Case 17, a 76-year-old male with RCC (arrow) of right femur treated by pedicle freezing. **a**. Radiograph of preoperative X-ray. **b**, **c**. CT and MRI scan. **d**. Joint dislocation and exposure of proximal femur after all of soft tissue were detached. **e**. Resection of biopsy tracts and curettage of bone tumor. **f**. Thawing at room temperature. **g**. reconstruction by frozen autograft combination with prosthesis and bone cement. **h**. Radiograph after immediate operation. **i**. Radiograph after operation 61 months

Basically, according to the radiological examination, patients with osteoblastic tumor or destruction of less than 1/3 of the cortical bone were given the frozen autograft only treatment for reconstruction. In case with large destruction of cortical bone, bone cement or bone graft were augmented in the bone defect. The lateral approach with curettage of the tumor was used in all patients and biopsy tracts were widely excised with the surgical margin. Any extraskeletal masses surrounding the lesions are resected to obtain adequate surgical margin. Following joint dislocation, the gluteus medius were peeled from greater trochanter, and maintain the continuity of the tensor fascia lata. Afterwards, all the muscles and tendons were detached from proximal femur until the proximal femur can be completely rotated and immersed in liquid nitrogen. To prevent frozen graft fracture, bone marrow and tumor were removed from medullary cavity. The surrounding normal soft tissues are protected by surgical sheets before immersing the proximal femur immersed into the liquid nitrogen. Then, additional 2 cm bone away from the margin of bony lesion was insert in liquid nitrogen by the pedicle freezing method for 20 min, thawing at room temperature for 15 min and in distilled water for 15 min. A tourniquet was used to prevent bleeding and tumor dissemination during the pedicle freezing [9] **(**Fig. 1**)**. An intertrochanteric osteotomy was performed, and the greater trochanter was preserved. The bipolar hemiarthroplasty, cements (antibiotic and anti-tumor drugs were included), long-stemmed prosthesis (span the frozen area) were used in reconstruction **(**Fig. 2**)**. Finally, the gluteus medius muscles was re-attached to their original anatomical sites using braided polyblend polyethylene suture (bone to tendon). Postoperatively, functional exercise (isometric exercise) was performed immediately from day 1. Full weight-bearing was permitted at 6 weeks post operation.

### Outcome measurement

All patients underwent clinical and radiological examinations at follow-up. Prosthetic failure was defined as removal of the original prosthesis for any cause. Bone absorption was defined as a lucent shadow around the autograft bone by radiological examination. The follow-up period was every 6 weeks to 3 months in years 1–2 after surgery, every 6 months in years 2–5 years, and every 6–12 months thereafter. Routine examinations included anteroposterior and lateral radiography, chest CT, and a bone scan or MRI as necessary.

### Statistical analyses

Patients functional outcome was evaluated using the MSTS functional score. The survival analysis was performed using the Kaplan-Meier method. The analyses were performed using SPSS ver. 24.0(IBM®).

## Results

**Fig. 3:**
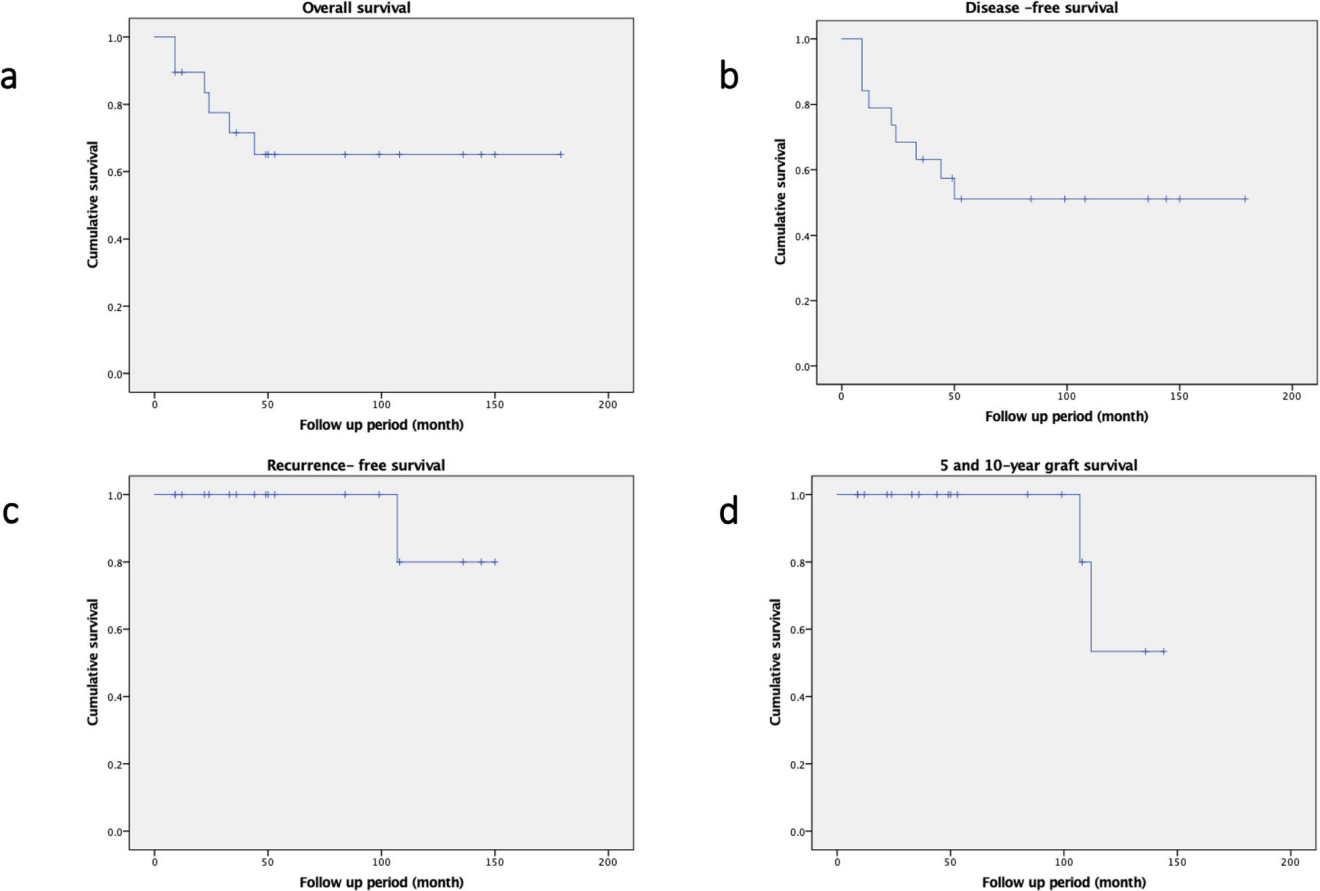
Kaplan-Meier curve of overall survival (**a**). Kaplan-Meier curve of the disease-free survival (**b**). Kaplan-Meier curve of the recurrence-free survival (**c**). Kaplan-Meier curve of the graft 5- and 10-year survival rates (**d**)

The average prosthetic length of the femoral component was 224 mm (range, 100–335 mm), while mean length of freezing area was 138 mm (range, 50–270 mm). The overall survival rate was 68.4%, and the disease -free survival rates were 50 and 50% at 5 and 10 years respectively **(**Fig. 3a, b**)**. Five-year and 10-year recurrence rates were 100 and 80%, respectively **(**Fig. 3c). At the last follow-up, 6 of 19 patients were continuous disease free, 3 were alive with disease, 4 had no evidence of disease after treatment for metastasis or recurrence, and 2 patients had lung metastasis for which they underwent thoracoscopic excision. 1 patient had bone metastasis of lung cancer that was treated by wide resection. One other patient had local recurrence of disease from residual soft tissue around the femur and underwent re-resection. Five patients died of the disease. The mean MSTS functional score of the patients was 26.4 points (rang, 19–30 points).

Among the 4 patients with the follow-up period > 10 years, the FAPC survival rates were 100 and 50% at 5 and 10 years respectively **(**Fig. 3d). Five of the 19 (26%) patients had complications, including 1 who developed a fracture at 12 months post operation, which had been controlled by conservative treatment. At 112 months post operation, the prosthesis broke, and the patient was subjected to conversion to megaprosthesis. Another patient developed local recurrence of the disease from residual soft tissue around the proximal femur for which hip disarticulation was performed at 107 months post operation (Table 1).

Two cases of deep infection around the acetabulum were managed with debridement, antibiotic bone cement implantation, and acetabular component revision. Wear of the acetabulum occurred in 2 cases, both of which were treated by conversion to an acetabular component at 97 and 106 months postoperatively. One patient had a dislocation that was managed by using closed reductions. At the last follow-up, no patients had greater trochanter avulsion, obvious absorption around the frozen bone, prosthetic loosening, or leg length discrepancy.

## Discussion

With the collaboration of multidisciplinary team, the life expectancy has been increased significantly in patients suffer from malignant tumor. Therefore, to improve the quality of life of patients, various reconstruction methods after bone tumors excision have been developed, including megaprosthesis, allografting, and tumor-bearing bone grafts (irradiated bone, pasteurized bone, and frozen bone). Given that biological reconstructions can achieve acceptable long-term functional outcome, biological reconstructions have received increasing attention [10].

Tumor-bearing bone graft is one of the biological reconstructions. In the past 20 years, tumor-bearing autografts frozen with liquid nitrogen have been reported as safe and effective methods for treating osteoblast tumors of various types and locations in basic experimental studies and clinical practices [8, 11–15]. The beneficial effects include a shorter union period, restoration of bone stock, lower cost, osteoinduction, osteoconduction, perfect fit, ease of soft-tissue attachment, activation of antitumor immune response and decreased disease transmission [8, 12]. In fact, FAPC by pedicle method shows significant advantage in proximal femoral tumors. First, it does not require femoral osteotomy or wait for the junction healing. Second, it is easy to reattach the ligaments and soft tissue around the proximal femur to the original anatomic site to increased hip joint stability. Third, it can potentially preserve maximal bone matrix to avoid further retreatment resulting from insufficient bone mass. In theory, all biological reconstructions have similar advantages and disadvantage. However, due to the loss of osteoinductive and osteogenic properties after thermal or radiation treatment, the allograft might have potential risk factors which require further surgery, such as nonunion with the host bone, graft fracture, bone resorption and immunological reactions [5–7]. Takata et al. also reported that tumor-bearing frozen bones maintains their microstructure and osteoinductive ability compared to pasteurization, autoclaving and allograft [11].

Biomechanical stability is of great concern to biological reconstructions. Lee et al. reported that the pasteurization decreases the biologic and mechanical properties and reduces strength to less than that of an allograft [16]. Interestingly, Yamamoto et al. had reported that the frozen bone has sufficient biomechanical strength for limb reconstruction that is comparable to pasteurized autografts and allografts [13].

Previous studies reported that an APC reconstruction exhibited satisfactory 5-year survival rate (72–90%) and MSTS score (77–90%), respectively. APC is a better reconstruction option if easily available. Remarkably, graft–host junction union is a major problem, and the nonunion rate is reportedly 5–19% [5, 6, 17–19]. On the other hand, Eid et al. [20] reported the outcomes of the application of the pasteurized APC in 18 patients; MSTS functional score was 80%, 5- and 10-year graft survival rates of 86%, respectively; mean graft-host junction union time of 13 months, and 1 case of non-union. Another study using extracorporeal irradiated APC by Chen et al. [21] reported an MSTS score of 72%, mean graft-host junction union time of 20 months, and 5-year graft survival rate of 85%. It seems that all biological reconstruction methods feature acceptable functional outcomes and implant survival rates. A few cases of nonunion or delayed union have been reported, although many studies reported that using the step-cut osteotomy, autogenous or allogenous bone graft into host bone, and non-cemented prostheses increased graft-host junction healing and stability in all biological reconstructions [5, 19, 22] **(**Table 2).

**Table 2:** Published studies of biological reconstruction and megaprosthesis replacement

Author	Method	Year of publication	Number of patients	MSTS score	Non-union of junction rate	Union time (mon)	follow-up period (year)	5 years Graft Survival(%)	10 years Graft Survival(%)	Infection(%)	Recurrence(%)	Graft Fracture(%)	Loosening	Dislocation
Menendez LR. et al. [4]	Megaprosthesis	2006	96	73%	N/A	N/A	1.5 (0.1–11)	82%	82%	6%	3%	N/A	0%	10%
Zehr RJ. et al. [17]	Megaprosthesis	2003	18	80%	N/A	N/A	9.5(0.3–23)	65%	58%	6%	12%	N/A	6%	6%
Finstein HL.et al. [24]	Megaprosthesis	2007	62	71%	N/A	N/A	5(0.1–22)	79%	57%	5%	7%	N/A	10%	5%
Iyas l. et al. [25]	Megaprosthesis	2002	15	63%	N/A	N/A	6.7(3–10)	Not report	Not report	13%	7%	N/A	7%	20%
Dubory A. et al. [16]	APC	2017	30	77%	16%	Not report	14.7(6.3–32.6)	73.00%	54%	4%	7%	13%	4%	9%
Lanlais F.et al. [5]	APC	2003	21	77%	11%	Not report	5.7(0.25–15)	90%	81%	0%	29%	0%	14%	0%
Muscolo DL. et al. [18]	APC	2010	38	90%	7%	Not report	7.5(3–17)	72%	69%	7%	3%	18%	0%	0%
Biau DJ. et al. [6]	APC	2010	32	Not report	19%	Not report	5.7(0.2–19.3)	86%	81%	13%	9%	3%	6%	3%
Chen CF. et al. [20]	Irradiation	2009	14	72%	8%	20 (14–40)	5.6 (1.5–9.75)	85%	Not report	0%	0%	0%	0%	0%
Eid AS. et al. [19]	Pasteurization	2011	18	80%	5%	13(6~18)	7.75(1.17–13.6)	86%	86%	11%	16%	0%	6%	6%
Current study	FAPC		19	87.60%	N/A	N/A	5.2 (0.67–15)	100%	50%	5%	5%	5%	0%	5%

*APC* allograft-prosthesis composite, *FAPC* frozen autograft-prosthesis composite, *N/A* not available

Greater trochanter stability in biological reconstruction is also a concern. Instability of abductors reconstruction prone to potentially severe complications, such as greater hip abductors avulsion, resorption, hip abductor avulsion, Trendelenburg gait or dislocation, which lead to poor function and a protracted postoperative rehabilitation period [19, 20]. Although abductors reconstruction methods are controversial, the hip abductor strength and gait which APC seems to be superior to megaprothesis [5, 19, 23]. A few studies emphasize the importance of preserving the proximal capsule and re-sutured onto the allograft to prevent hip dislocations [17]. In the current study, similar procedures were also applied to peeled gluteus medius from greater trochanter and reattached the gluteus medius to the original site. Bone to tendon reconstruction which might easy to restore abduction strength due to integrity of the gluteus medius. In this study, only one patient occurred joint dislocation, which is comparable to other methods of biological reconstruction [7, 23, 24].

In addition, bone cement or graft plus long-stem-prosthesis has been used in some patients with metastases combined lytic to make up for osteolytic destruction in the proximal femur. Such treatment will provide sufficient mechanical strength and stabilization of bones. Given that pedicle freezing required more a longer incision and muscle dissection, the authors encouraged isometric exercise to avoid complication in early postoperative period. The bone cement and prosthesis provide rigid fixation and avoid bone union which make it possible for full weight-bearing at 6 weeks post operation. Meanwhile, all the patients underwent physical exercise to learn prevention hip dislocation, and postoperative bracing provided sufficient support to minimize dislocations if necessary. In case of large resection of muscles, the mesh can be used to attach surrounding soft tissues, and abduction brace is used to prevent hip dislocation. Of note, if patients showed signs of severe destruction of cortex (> 2/3) in the proximal femur, megaprosthesis was recommended [12].

Megaprosthesis has satisfactory short- to medium-term outcome, early mobilization and weight-bearing, and short operative time; however, abductor muscles reattachment remains an issue need to be concerned. Accumulating evidence indicates using an artificial ligament to affix the megaprosthesis can promote soft-tissue reconstruction and achieve better joint stability and functional outcomes [25]. In fact, artificial ligament use is probably unable to reduce the occurrence of prosthetic complications such as aseptic loosening, prosthesis breakage, infection, and stress shielding [26]. Moreover, long-term prosthetic failure rate is between 6 and 33%, mean MSTS score is between 63 and 83%, and the major complications were infection (5–13%) and dislocation (0–20%) [4, 18, 26–28] **(**Table 2). The application of silver– or iodine– coated implants had been reported to reduce overall infection rates [29, 30]. However, a higher cost burden, unavailability in some countries and the limited bone mass complicate revision surgeries.

The various reconstructive alternatives have acceptable oncological and functional outcomes. However, each method has its own limitations, and thus it is crucial for choose the proper method to maximize the benefits for each patient. Surgeons must carefully consider the patient’s age, general condition, response to chemotherapy, and expectations when individualizing a treatment plan.

In the current study, the functional outcome was similar to those of other reconstruction methods. The mean MSTS functional score was 88%, and the 5- and 10-year graft survival rates were 100 and 50%, respectively. At the last follow-up, no prosthesis loosening or obvious lucent shadows around the autograft bone was observed on radiological examination. Only 1 patient had local recurrence around the residual soft tissue for which hip disarticulation was performed after 2 re-excisions. Wear of the acetabulum occurred in 2 patients despite the use of bipolar hemiarthroplasty, but this might be an inevitable long-term complication of joint replacement.

This study had several limitations. First, due to its retrospective design and single center, a relatively small number of patients were enrolled, a follow-up > 10 years was available for only 4 of the 19 patients. Second, no control group was available for comparison of functional outcomes; thus, our results could be compared to only those of prior studies. Similarly, the accuracy of our results was lower than those of randomized study. Third, the patients had various diagnoses and were treated with various chemotherapy regimens, which might affect survival rates and functional outcomes. Therefore, to assess the efficacy and safety of this procedure, a prospective study which compares the functional outcomes and survival rates of several reconstruction methods over a long-term follow-up period needs to be performed in the future.

## Conclusions

Our findings suggest that FAPC reconstruction is a good method of treating patients with malignant bone tumor in the proximal femur. With no femoral osteotomy, FAPC reconstruction can achieve a better clinical outcome and reduce complications, which is comparable to other methods of biological reconstruction or megaprosthesis.

## Data Availability

The datasets will be available from the corresponding author if need to obtain the data and materials.

## Abbreviations

APC: Allograft–prosthesis composite
FAPC: Frozen autograft–prosthesis composite
MSTS: Musculoskeletal Tumor Society
